# OR6-002 – Thromboembolism in autoinflammatory syndromes

**DOI:** 10.1186/1546-0096-11-S1-A97

**Published:** 2013-11-08

**Authors:** PM Vaitla, R Weldon, I Tynan, EM McDermott, E Drewe

**Affiliations:** 1 Immunology, Nottingham University Hospitals NHS Trust, Nottingham, UK

## Introduction

Venous thromboembolism (VTE) has been associated with FMF but risk has not been studied in other auto inflammatory syndromes. We therefore did a retrospective study of incidence of VTE in a cohort of patients under Nottingham University Hospitals.

## Objectives

To identify the incidence and site of VTE in patients with auto inflammatory syndrome managed at our centre from 1995 to 2013. In addition we sought to investigate risk factors for VTE present, including laboratory evidence of inflammation.

## Methods

The case records of patients with auto inflammatory syndrome were studied to assess history and site of VTE. Subtype of auto inflammatory disease was noted along with risk factors for VTE and therapy for auto inflammatory disease. In addition it was assessed if auto inflammatory disease had been clinically active at the time of VTE and whether inflammatory markers were elevated in the 6 months prior to VTE. Mean and median values for highest CRP in the 6 months preceding the episodes of VTE were calculated for the affected group.

## Results

45 patients with auto inflammatory disease were included in the study (22 with TRAPS, 2 with HIDS, 4 with FMF, 2 with CAPS, 1 with variant Schnitzler, 1 with NOD2 mutations and 13 undefined). 7 subjects (15.5% of cohort) had a total of 11 episodes of VTE. This comprised 3 with TRAPS, 1 with CINCA, 1 variant Schnitzler’s syndrome, 1 had auto inflammatory syndrome with NOD2 mutations and granulomas and one undefined under investigation. VTE included deep vein thrombosis (x 6), pulmonary embolism (x 4) and Budd-Chiari syndrome (x 1). In those with multiple VTE, recurrence of VTE occurred 1 to 2 months after discontinuation of anticoagulation.

Apart from underlying auto inflammatory disease no risk factors for VTE were identified. 3 patients had thrombophilia screen which was negative excepting one patient with low protein C presumed due to liver dysfunction. At the time of VTE, all 7 patients were on prednisolone and in addition 2 patients were on methotrexate, 1 on chlorambucil and 1 on anakinra.

All patients reported recurrent flares or continuous auto inflammatory symptoms prior to VTE. 6 out of 7 patients had raised CRP in the 6 months preceding VTE. CRP values in the 6 months preceding VTE were available for 10 episodes. The mean of the highest CRP in the 6 months preceding each VTE episode was 119.8, median was 124 with values ranging from 4 to 251.

3 patients are deceased. The 4 living patients are on lifelong warfarin. Of note pleuritic chest pain was initially attributed to flare of auto inflammatory disease and responded partially to methyl prednisolone delaying diagnosis of pulmonary emboli.

## Conclusion

In summary we report a high incidence of VTE in auto inflammatory disease, particularly in the setting of uncontrolled inflammation. VTE may mimic symptoms of auto inflammatory disease and needs to be considered to prevent diagnostic delay. This cohort includes patients managed prior to availability of biological therapies and now early introduction of biologicals to drive down acute phase response may reduce risk of VTE. We also propose whether VTE prophylaxis e.g. aspirin should be initiated for patients with uncontrolled inflammation despite therapy for auto inflammatory disease.

## Disclosure of interest

None declared

